# Minimally Invasive Plate Osteosynthesis for Closed Distal Tibial Fractures: Clinical and Radiographic Outcomes From a Three-Patient Case Series

**DOI:** 10.7759/cureus.108393

**Published:** 2026-05-06

**Authors:** Md Sulaiman, Md Shohel Rana

**Affiliations:** 1 Orthopaedic Surgery, Trauma General Hospital and Diagnostic Center, Brahmanbaria, BGD; 2 Orthopaedics and Traumatology, Trauma Center, Bhaluka, Mymensingh, BGD

**Keywords:** biological fixation, distal tibia fracture, distal tibial locking plate, extra-articular fracture, fracture healing, minimally invasive plate osteosynthesis, orthopedic trauma

## Abstract

Distal tibial fractures present a therapeutic challenge due to limited soft-tissue coverage and the need to achieve stable fixation while preserving local vascularity. Minimally invasive plate osteosynthesis (MIPO) has emerged as a biologically favorable technique for managing selected extra-articular distal tibial fractures by minimizing soft-tissue disruption and maintaining fracture hematoma. This case series describes three patients with right-sided closed extra-articular distal tibial fractures classified as AO Foundation/Orthopaedic Trauma Association (AO/OTA) 43-A1.2, managed with MIPO using a medial distal tibial locking plate with supplementary fibular fixation. All patients underwent early definitive fixation within hours of injury following initial stabilization. Postoperative protocols included staged weight-bearing progression guided by radiographic healing. All cases demonstrated satisfactory reduction and maintained alignment throughout follow-up, with progressive callus formation and eventual fracture union. No wound complications, implant failure, malalignment, or reoperation were observed. Functional outcomes were favorable, with good to excellent ankle function in all patients and American Orthopaedic Foot & Ankle Society (AOFAS) scores of 90 and 85 in the assessed cases. This series highlights that MIPO is an effective and reliable treatment option for selected extra-articular distal tibial fractures, providing stable fixation, preservation of fracture biology, and satisfactory clinical and radiographic outcomes when appropriate patient selection and surgical technique are applied.

## Introduction

Distal tibial fractures remain difficult injuries to manage because of the subcutaneous location of the bone, limited soft-tissue coverage, and the need to achieve stable fixation without further compromising local vascularity. In extra-articular distal tibial fractures, treatment must balance the restoration of alignment and stability with preservation of fracture biology, as wound problems, delayed union, malalignment, and infection remain important concerns [1-3].

Minimally invasive plate osteosynthesis (MIPO) has emerged as an attractive option for selected distal tibial fractures because it minimizes soft-tissue dissection, preserves periosteal circulation and fracture hematoma, and allows indirect reduction with stable fixation [4-6]. Previous reports have shown satisfactory union rates and acceptable complication profiles with MIPO, while comparative studies have suggested advantages in alignment control relative to some alternative fixation methods in selected extra-articular fracture patterns [5-7].

The present case series describes the clinical and radiographic outcomes of three patients with right-sided closed extra-articular distal tibial fractures classified as AO Foundation/Orthopaedic Trauma Association (AO/OTA) 43-A1.2, all treated with MIPO using a medial distal tibial locking plate with supplementary fibular fixation. This series highlights fracture selection, biological fixation principles, and short- to long-term radiographic and functional outcomes in a uniform subgroup of distal tibial fractures.

## Case presentation

Case 1

**Figure 1:**
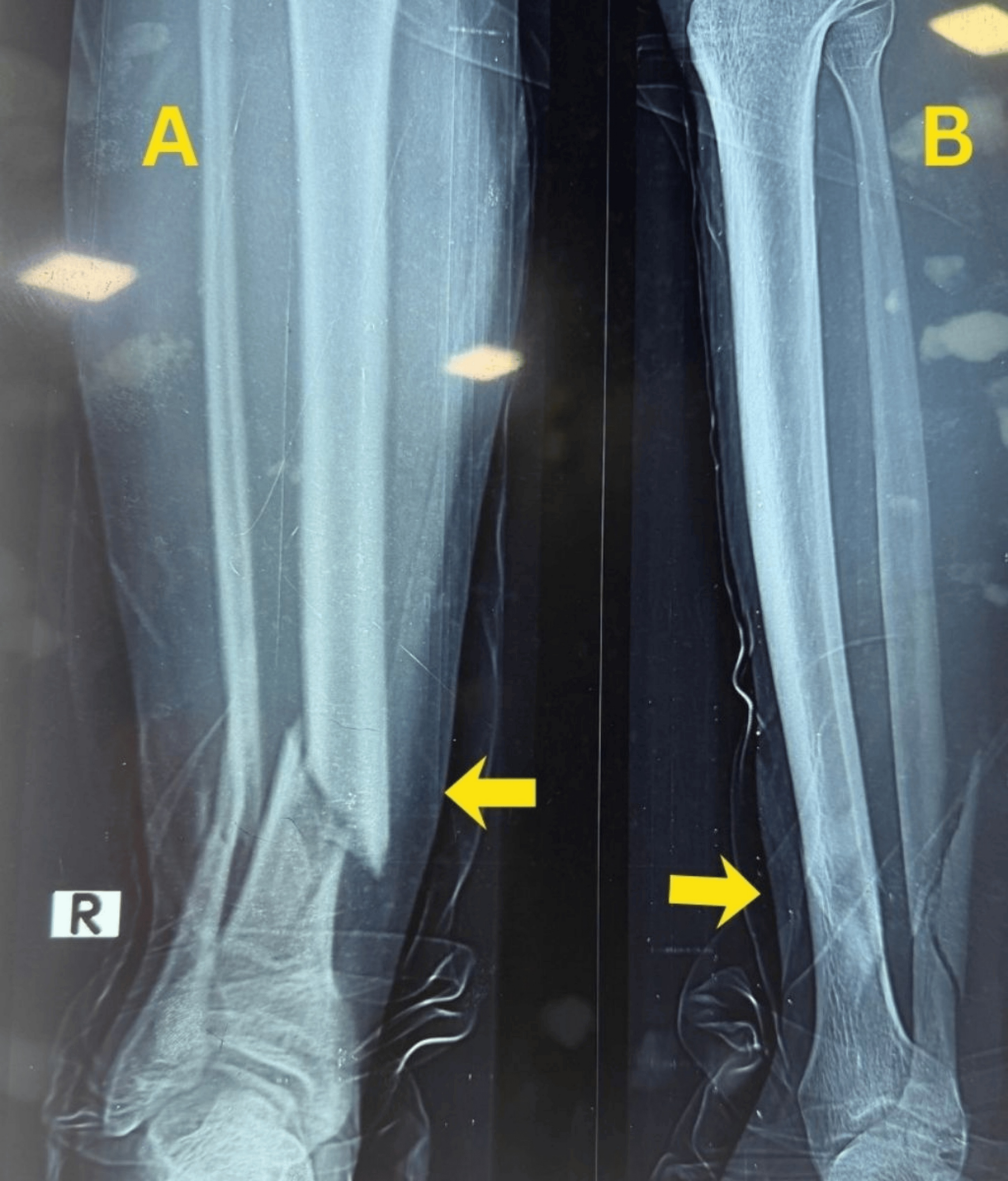
Preoperative radiographs of Case 1. (A) Anteroposterior view showing an extra-articular distal tibial fracture with an associated fibular fracture. (B) Lateral view demonstrating fracture configuration, displacement, and distal tibial involvement.

A 31-year-old man sustained a right closed distal tibia and fibula fracture following a road traffic accident and presented approximately 35 minutes after injury. The injury was classified as a closed fracture with Tscherne grade I soft-tissue involvement. Initial stabilization was performed with temporary plaster immobilization, and preoperative ankle range of motion was preserved. Plain anteroposterior and lateral radiographs demonstrated an extra-articular distal tibial fracture classified as AO/OTA 43-A1.2, with a short oblique tibial fracture pattern and an associated comminuted fibular fracture with a large third fragment (Figure 1).

There was no radiographic evidence of tibial plafond involvement.

**Figure 2:**
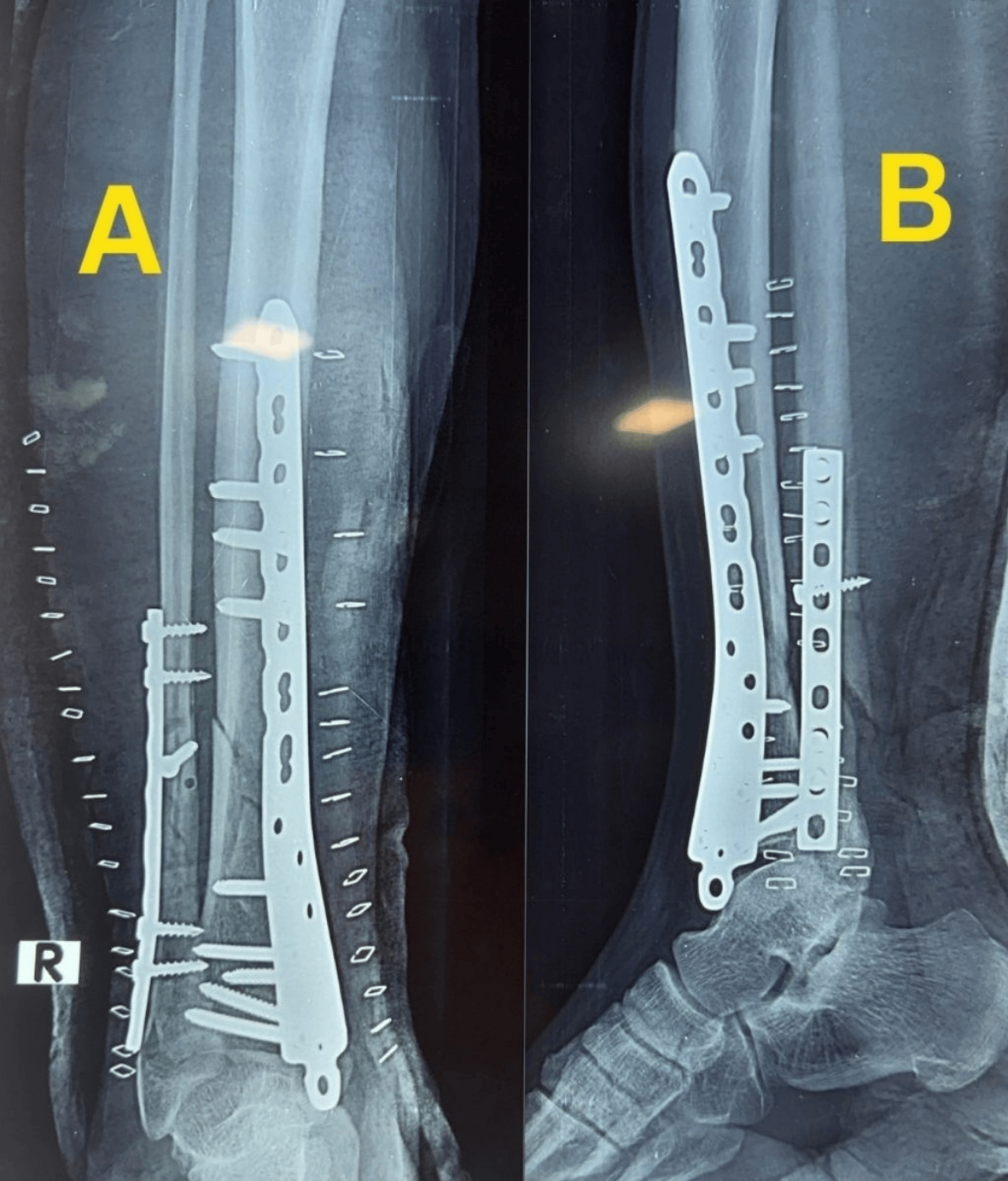
Figure 2. Immediate postoperative radiographs of Case 1. (A) Anteroposterior view showing fixation with a medial distal tibial locking plate and fibular plate with satisfactory alignment. (B) Lateral view confirming stable fixation and restoration of alignment.

Definitive fixation was carried out on the day of admission, approximately five hours after injury, using MIPO with a medial distal tibial locking plate and supplementary fibular plating. Immediate postoperative radiographs demonstrated satisfactory reduction, restoration of alignment, and stable fixation (Figure 2).

**Figure 3:**
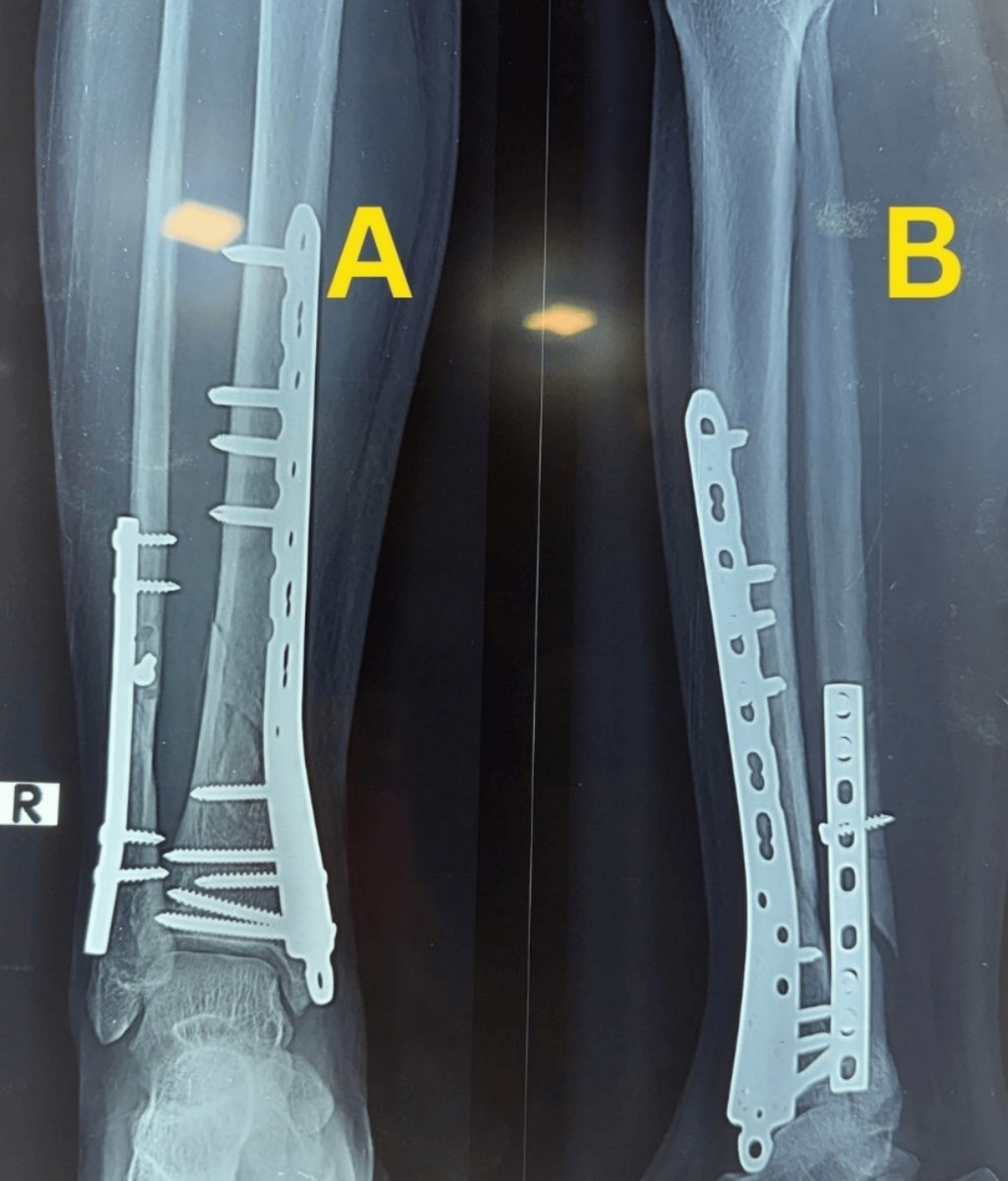
Early follow-up radiographs of Case 1. (A) Anteroposterior view demonstrating maintained alignment with early callus formation at the fracture site. (B) Lateral view showing progression of healing without loss of reduction.

Partial weight bearing was initiated at six weeks and progressively advanced according to radiographic callus formation, with full weight bearing achieved by 10 weeks. Early follow-up radiographs demonstrated maintained alignment with early callus formation and no loss of reduction (Figure 3).

**Figure 4:**
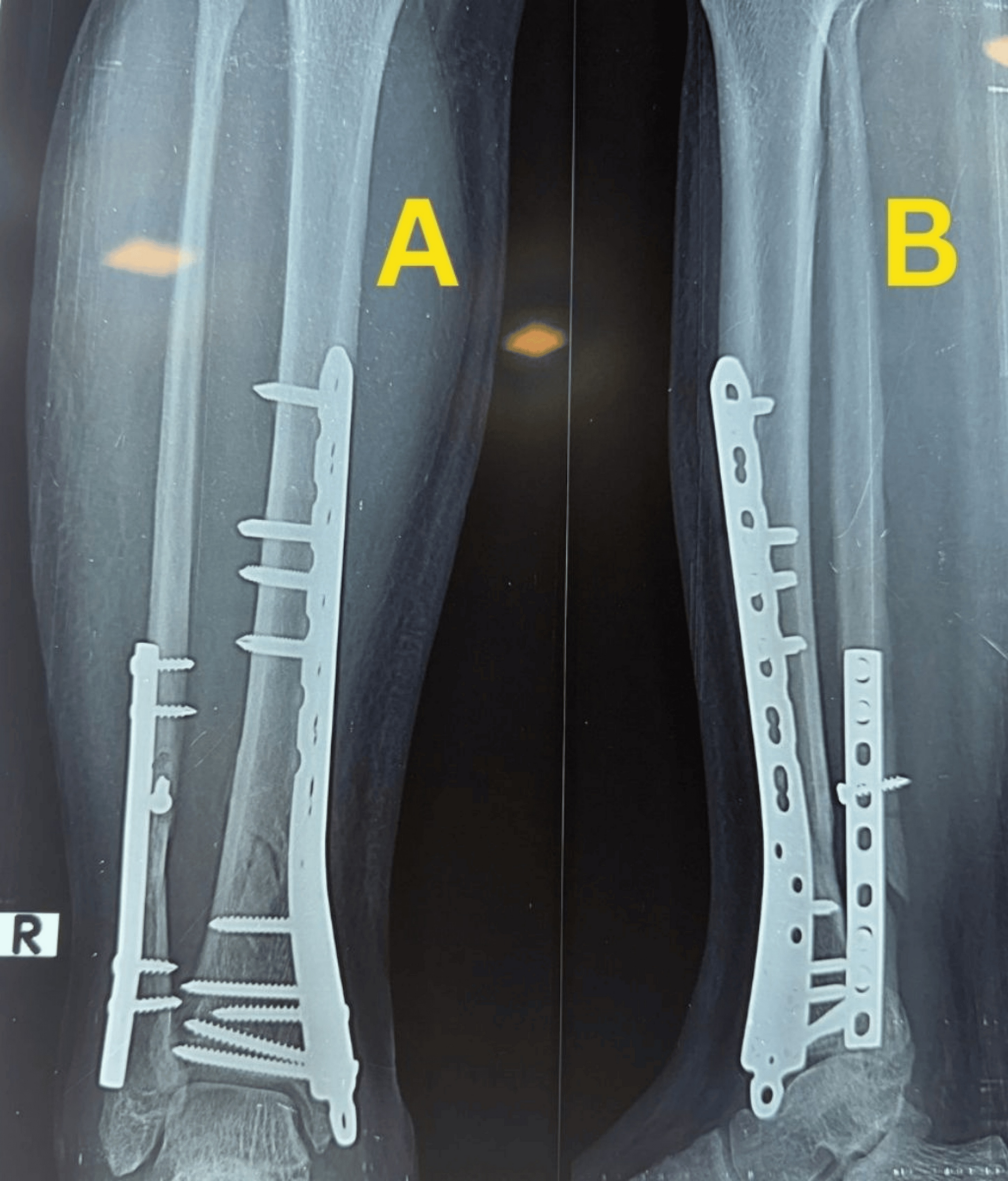
Final follow-up radiographs of Case 1. (A) Anteroposterior view showing complete fracture union with good alignment and remodeling, with the implants in situ. (B) Lateral view confirming solid union of the distal tibia.

Final follow-up radiographs showed complete fracture union with good alignment and remodeling, with the implants in situ (Figure 4).

**Figure 5:**
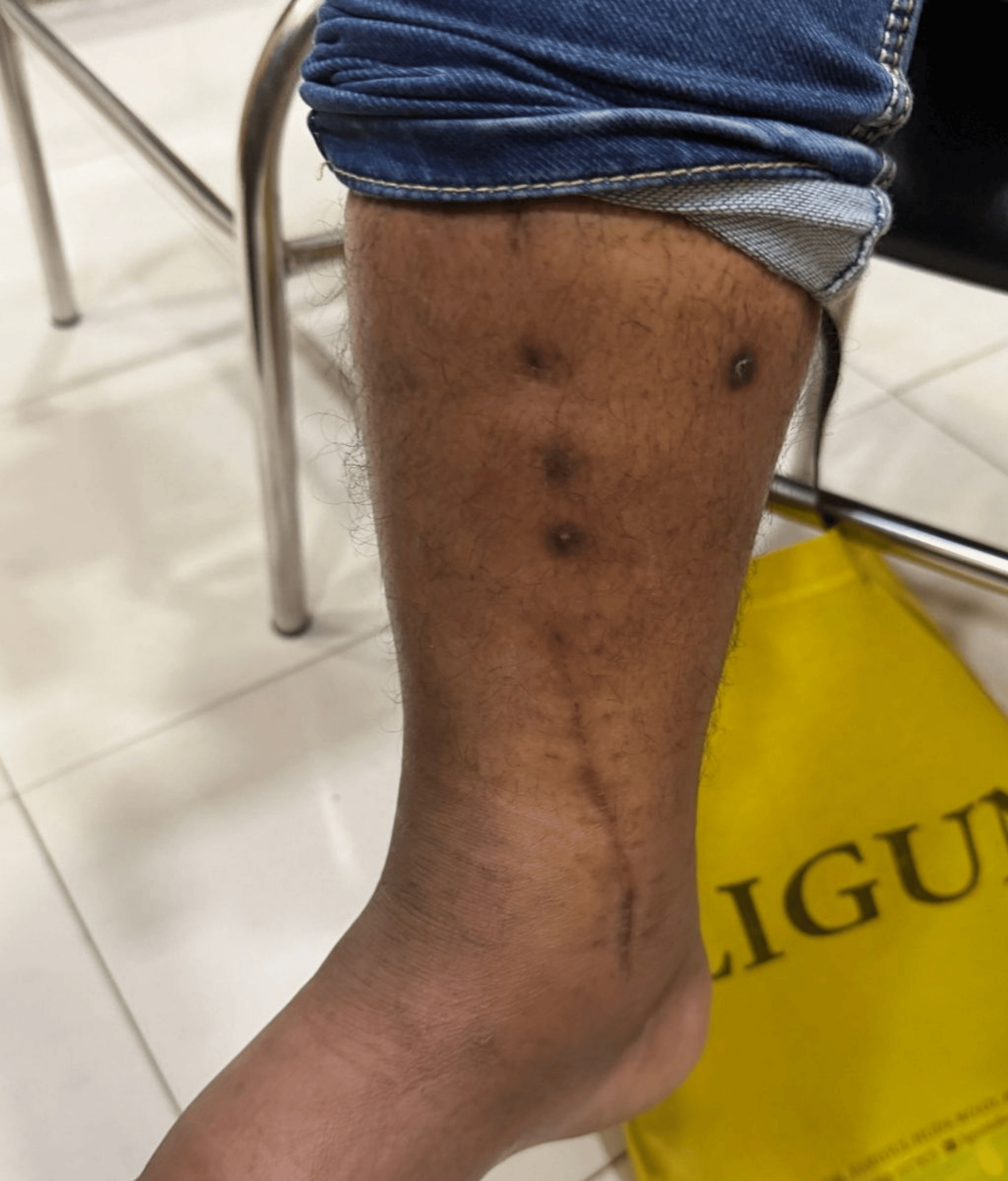
Clinical outcome of Case 1. Clinical photograph at the final follow-up, showing a well-healed surgical scar with a satisfactory soft-tissue condition.

Clinically, the patient had a well-healed surgical scar with satisfactory soft-tissue condition at the final follow-up (Figure 5).

At the final follow-up, the patient demonstrated near-normal ankle function, and the American Orthopaedic Foot & Ankle Society (AOFAS) ankle-hindfoot score was 90, indicating an excellent functional outcome.

Case 2

**Figure 6:**
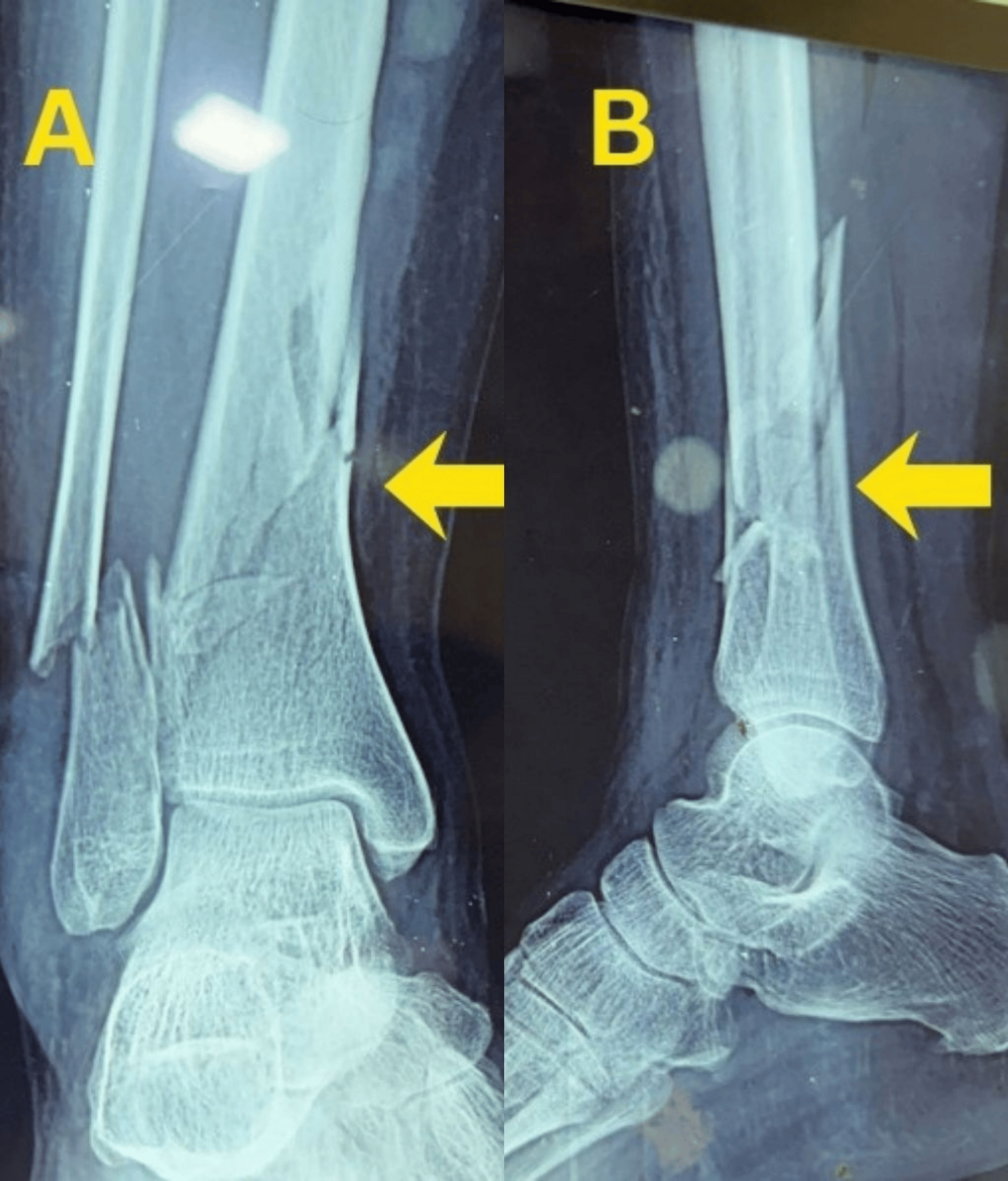
Preoperative radiographs of Case 2. (A) Anteroposterior view showing an extra-articular distal tibial fracture with an associated fibular fracture. (B) Lateral view demonstrating short oblique fracture morphology and displacement.

A 50-year-old woman sustained a right closed distal tibia and fibula fracture following a fall and presented approximately one hour after injury. The injury was classified as a closed fracture with Tscherne grade 0 soft-tissue involvement. Initial stabilization was performed with temporary plaster immobilization. Plain anteroposterior and lateral radiographs demonstrated an extra-articular distal tibial fracture classified as AO/OTA 43-A1.2, with short oblique fracture morphology involving both the tibia and fibula (Figure 6).

There was no radiographic evidence of tibial plafond involvement.

**Figure 7:**
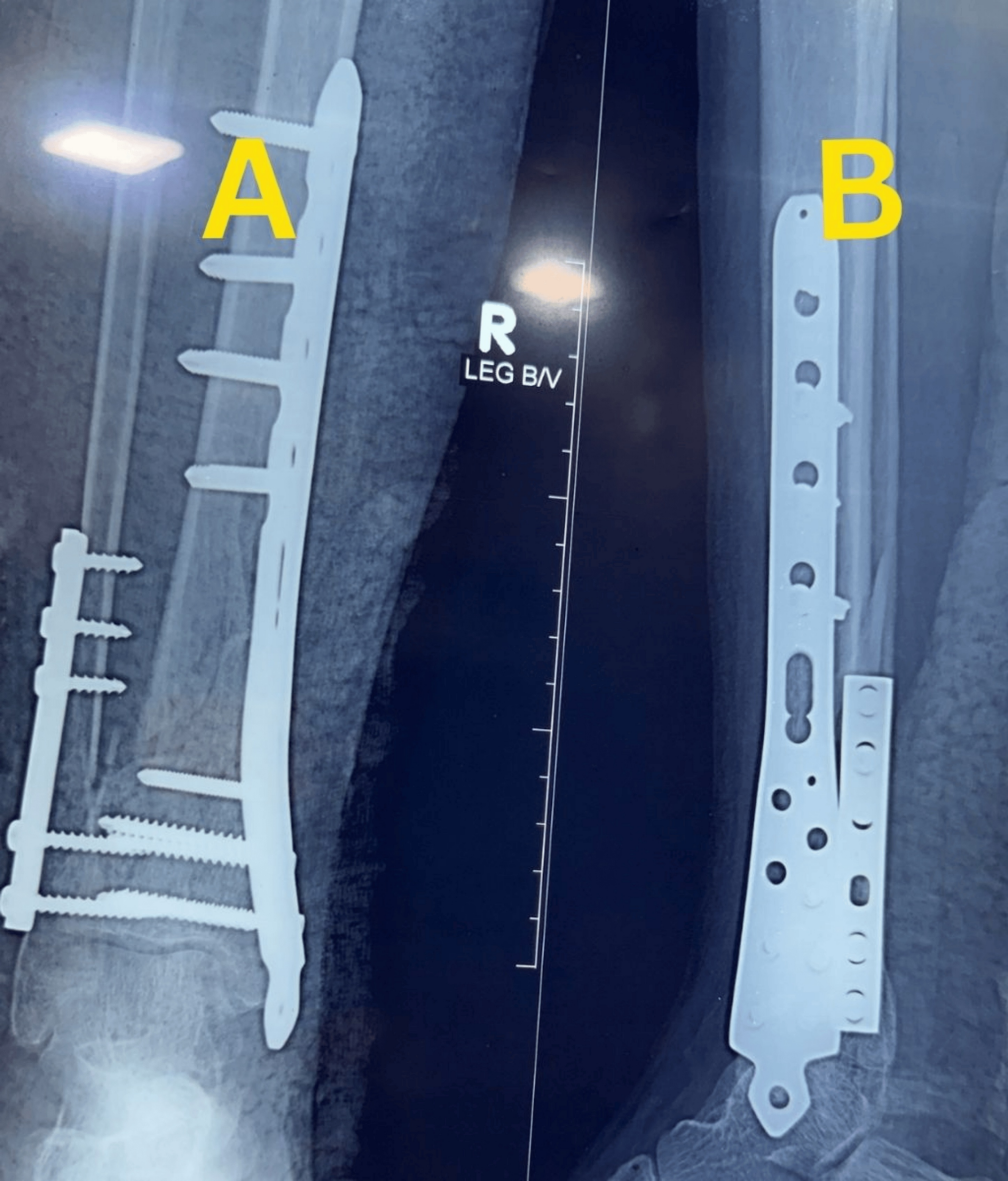
Immediate postoperative radiographs of Case 2. (A) Anteroposterior view showing fixation with a medial distal tibial locking plate and fibular plate with satisfactory alignment. (B) Lateral view confirming stable fixation and restoration of anatomical alignment.

Definitive fixation was carried out approximately five hours after injury using MIPO with a medial distal tibial locking plate and supplementary fibular plating. Immediate postoperative radiographs demonstrated satisfactory reduction, restoration of anatomical alignment, and stable fixation (Figure 7).

**Figure 8:**
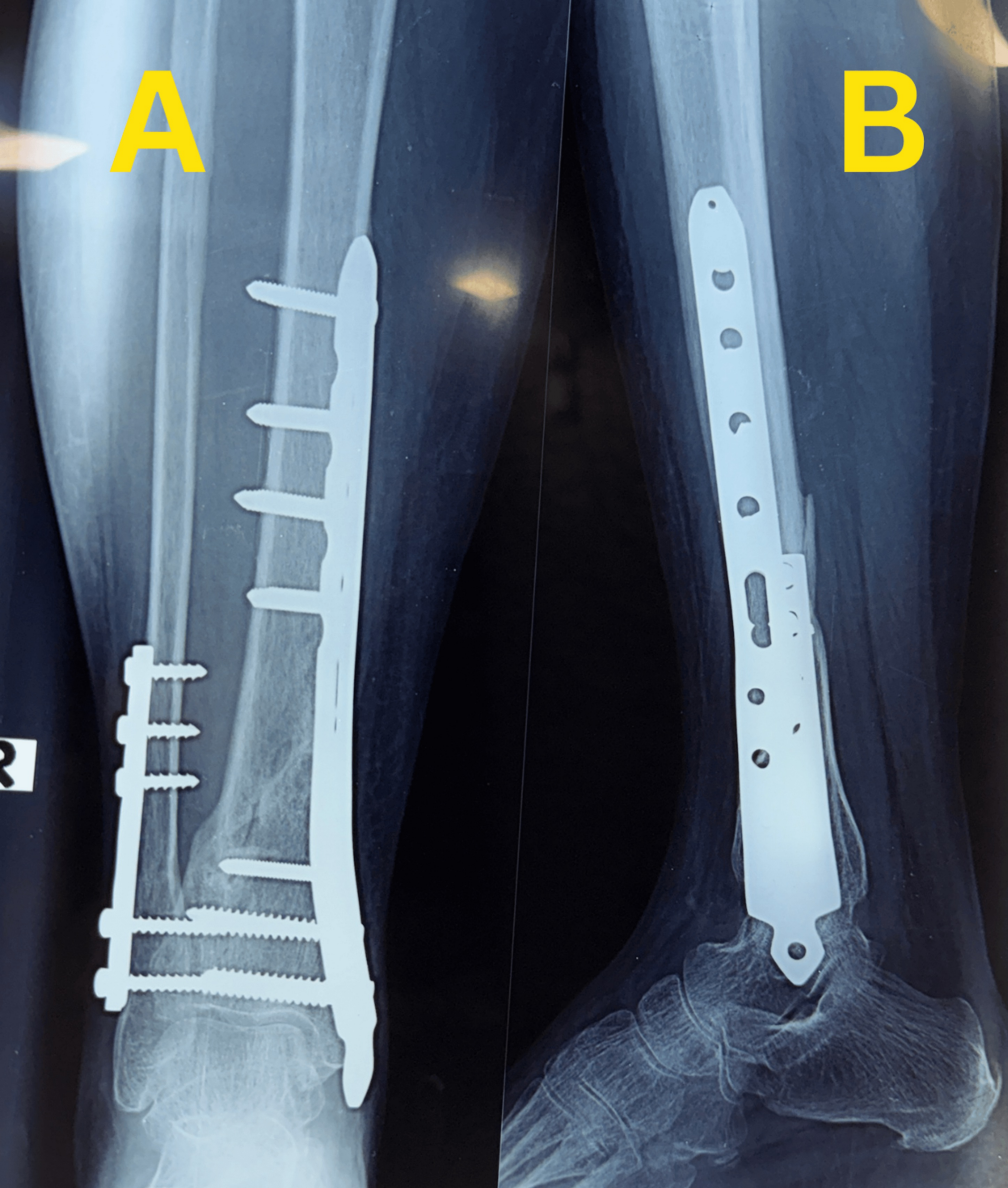
Follow-up radiographs during healing of Case 2. (A) Anteroposterior view demonstrating maintained alignment with visible callus formation at the fracture site. (B) Lateral view showing progressive fracture healing without implant failure or loss of reduction.

Partial weight bearing was initiated at six weeks and progressed according to radiographic healing, with full weight bearing achieved by 12 weeks. Follow-up radiographs during healing demonstrated maintained alignment with visible callus formation at the fracture site and progressive fracture healing without implant failure or loss of reduction (Figure 8).

**Figure 9:**
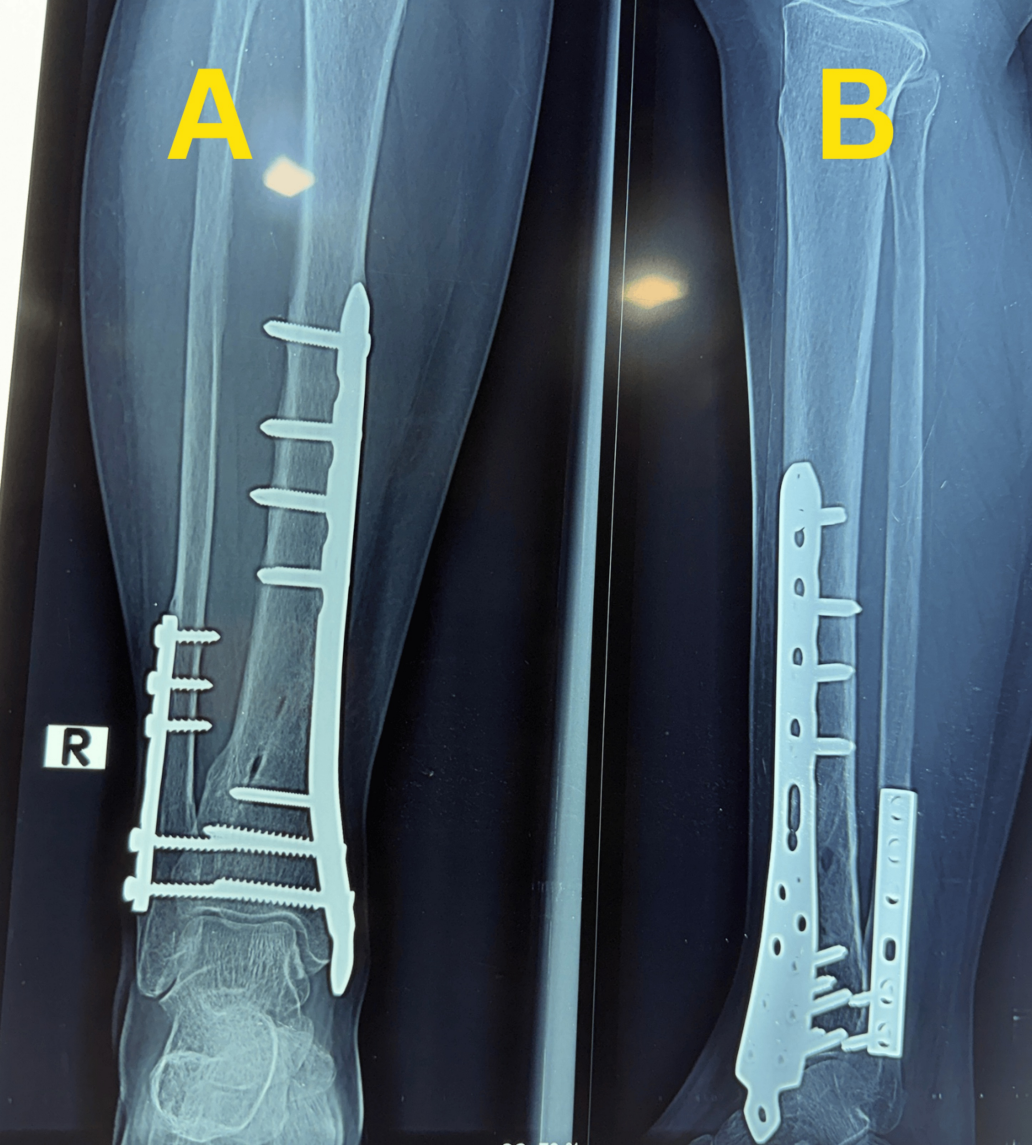
Final follow-up radiographs of Case 2. (A) Anteroposterior view showing complete fracture union with good alignment and remodeling of the distal tibia, with the implant in situ. (B) Lateral view confirming solid union and maintained alignment.

The final follow-up radiographs showed complete fracture union with good alignment and remodeling of the distal tibia, with the implant in situ (Figure 9).

At the final follow-up, the patient achieved a good functional outcome, with an AOFAS ankle-hindfoot score of 85.

Case 3

**Figure 10:**
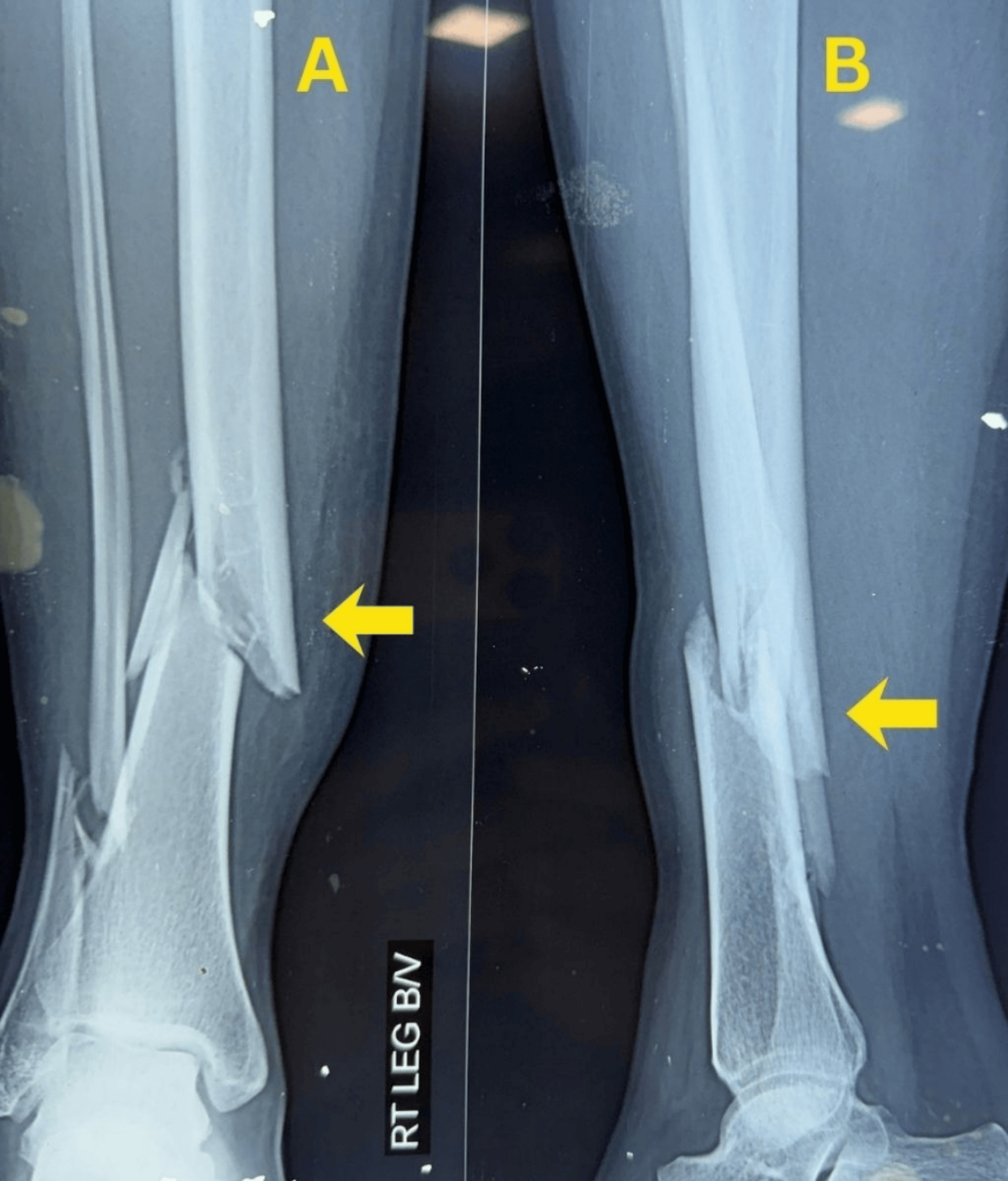
Preoperative radiographs of Case 3. (A) Anteroposterior view of the right distal tibia showing an extra-articular distal tibial fracture with associated fibular fracture. (B) Lateral view demonstrating fracture configuration, displacement, and distal tibial involvement.

A 44-year-old woman sustained a right closed distal tibia and fibula fracture following a fall and presented approximately one hour after injury. The injury was classified as a closed fracture with Tscherne grade 0 soft-tissue involvement. Initial stabilization was performed with temporary plaster immobilization. Plain anteroposterior and lateral radiographs demonstrated an extra-articular distal tibial fracture classified as AO/OTA 43-A1.2, with a short oblique configuration and mild comminution of the tibia, along with a short oblique fibular fracture (Figure 10).

There was no radiographic evidence of tibial plafond involvement.

**Figure 11:**
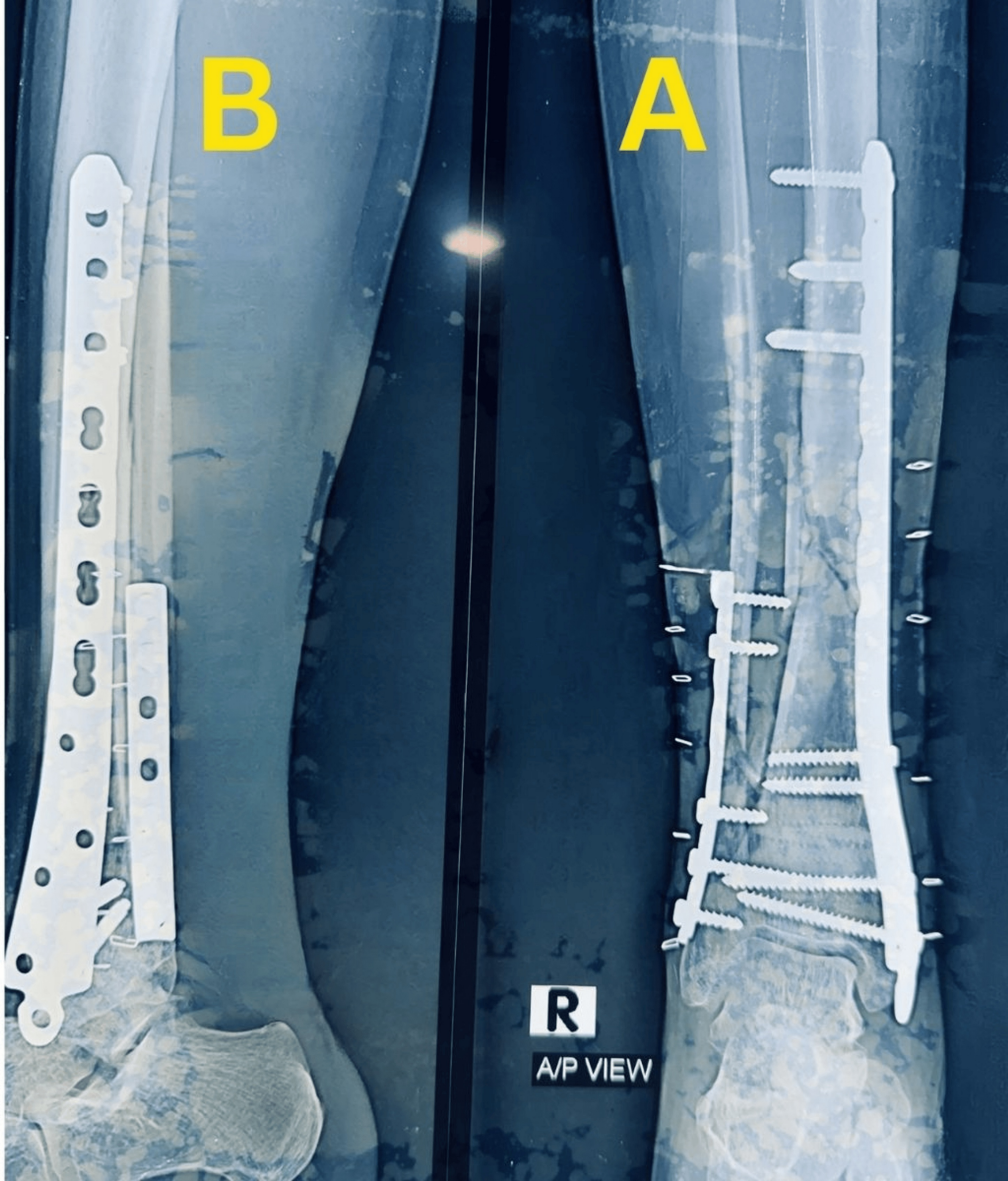
Immediate postoperative radiographs of Case 3. (A) Anteroposterior view showing fixation with a medial distal tibial locking plate and fibular plate with satisfactory alignment. (B) Lateral view confirming stable fixation and restoration of alignment.

Definitive fixation was carried out approximately five hours after injury using MIPO with a medial distal tibial locking plate and supplementary fibular plating. Immediate postoperative radiographs demonstrated satisfactory reduction, restoration of alignment, and stable fixation (Figure 11).

**Figure 12:**
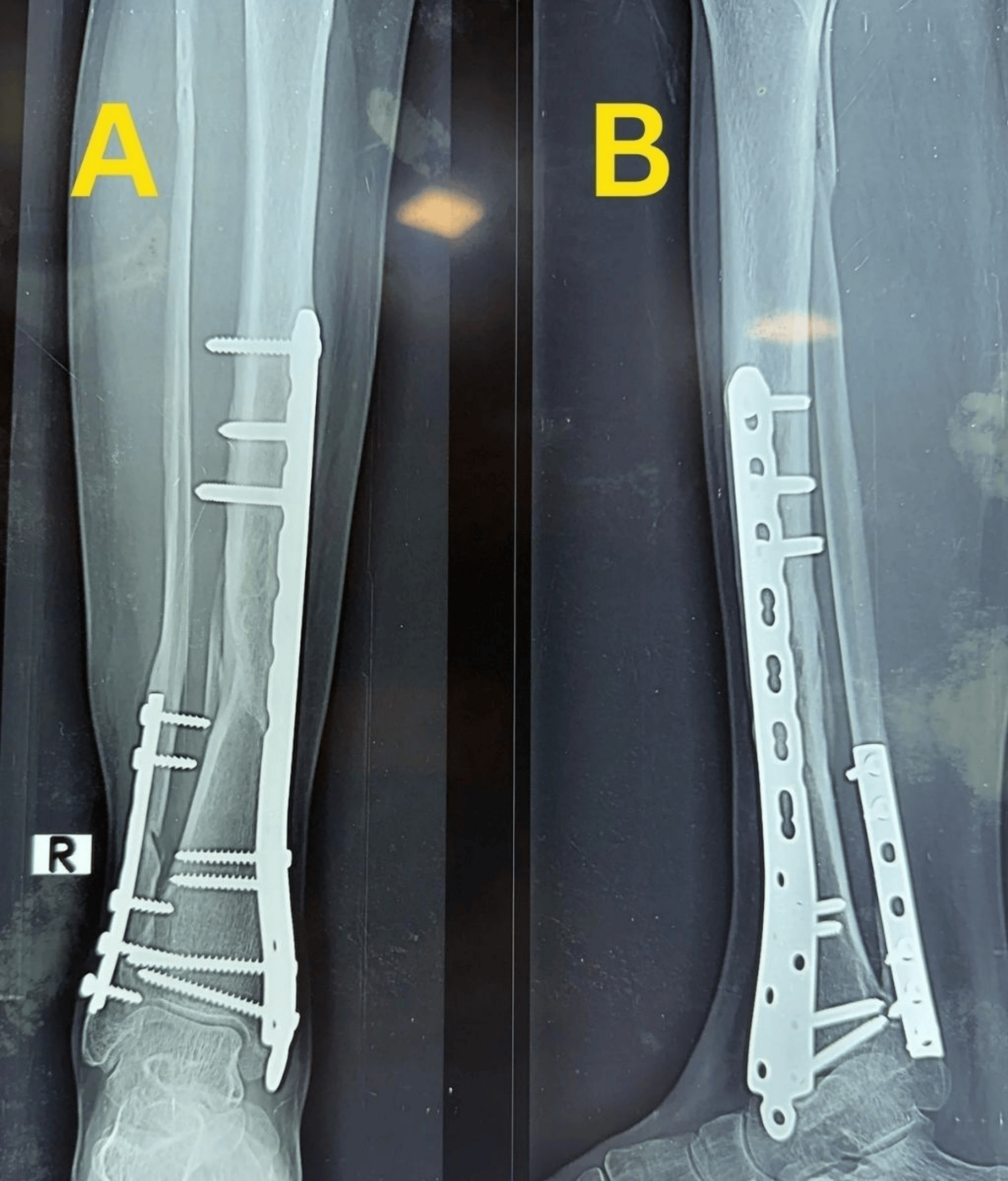
Follow-up radiographs showing fracture healing in Case 3. (A) Anteroposterior view demonstrating maintained alignment with bridging callus formation at the fracture site. (B) Lateral view showing progressive healing without implant loosening or loss of reduction.

Serial follow-up radiographs demonstrated progressive fracture healing. Follow-up radiographs showed maintained alignment with bridging callus formation at the fracture site and no evidence of implant loosening or loss of reduction (Figure 12).

**Figure 13:**
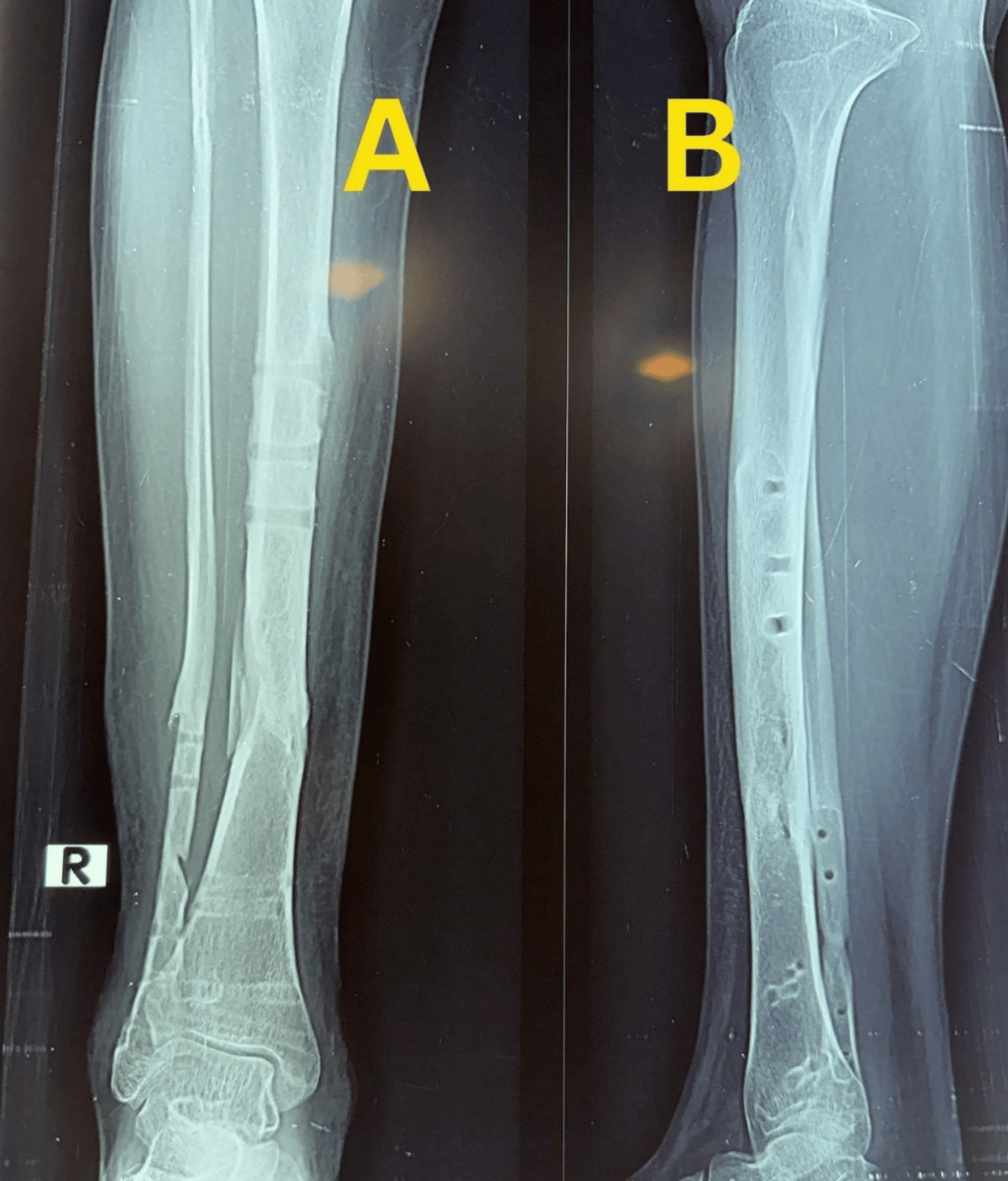
Radiographs after implant removal in Case 3. (A) Anteroposterior view after implant removal showing complete union and remodeling of the distal tibia. (B) Lateral view confirming maintained alignment and consolidated healing following hardware removal.

Subsequent radiographs obtained after implant removal confirmed complete union with remodeling, maintained alignment, and healed fracture morphology (Figure 13).

The precise timing of radiological union could not be established because serial follow-up radiographs were not obtained at uniform intervals. Nevertheless, subsequent radiographs demonstrated complete union with remodeling.

This retrospective case series was conducted in accordance with institutional ethical standards and the Declaration of Helsinki. Formal IRB/ethics committee approval was not required at our institution. Written informed consent for treatment and publication was obtained from the patients, and all identifying information has been removed.

## Discussion

Distal tibial fractures are difficult injuries to manage because the distal tibia is subcutaneous, has limited soft-tissue coverage, and is particularly vulnerable to further compromise of its blood supply during fixation. These factors increase the risk of wound complications, infection, delayed union, and malalignment after surgery [1-3]. The challenge is especially relevant in extra-articular distal tibial fractures, where stable fixation must be achieved while preserving fracture biology as much as possible [1-3].

MIPO was developed to address these concerns by limiting surgical dissection, preserving periosteal circulation, and maintaining the fracture hematoma while still allowing restoration of length, alignment, and rotation through indirect reduction techniques [4-6]. In distal metaphyseal fractures, this biological fixation strategy is particularly appealing because conventional open plating may further compromise already vulnerable soft tissues [2,4-6].

Previous studies have shown that MIPO can provide high union rates and satisfactory functional outcomes in distal tibial fractures, with acceptable rates of infection and soft-tissue complications when careful technique is used [5-7]. Comparative studies have also suggested that, in selected extra-articular distal tibial fracture patterns, MIPO may offer better control of alignment than intramedullary nailing, although both treatment options remain valid depending on fracture morphology, soft-tissue condition, and surgeon experience [7-10].

The present case series adds to this body of evidence by demonstrating consistent results in three right-sided extra-articular distal tibial fractures classified as AO/OTA 43-A1.2. Although there was some variation in patient age, mechanism of injury, and fracture morphology, all three cases were managed according to the same core principle: early biological fixation with minimally invasive medial distal tibial plating, supplemented by fibular fixation. In all patients, satisfactory reduction and alignment were achieved intraoperatively and maintained throughout follow-up, with no wound complication, implant failure, malalignment, or reoperation recorded during the observed period [4,7,8,11].

An important strength of this series is the relative uniformity of the fracture subgroup. All three fractures were extra-articular distal tibial injuries without radiographic plafond extension, making the series more internally consistent and allowing more meaningful comparison of outcomes. Because there was no radiographic evidence of plafond involvement, CT scanning was not required, and treatment planning could reasonably be based on plain radiographs alone. This homogeneity strengthens the practical message of the series: selected AO/OTA 43-A1.2 fractures appear well suited to MIPO when soft tissues are acceptable, and reduction can be achieved indirectly [1,2,4,7].

Another notable aspect is the timing of fixation. In all three patients, definitive surgery was performed approximately five hours after injury, following initial temporary immobilization and assessment. In carefully selected closed distal tibial fractures, early fixation may help prevent progressive swelling, facilitate indirect reduction, and simplify soft-tissue handling, although such an approach should always be individualized according to the condition of the soft tissues and the overall status of the patient [2,3,6]. In the present series, early stabilization was associated with satisfactory alignment, uneventful soft-tissue recovery, and successful fracture healing in all cases.

Fibular fixation was used in all three cases as an adjunct to tibial stabilization. Although the routine need for fibular fixation in distal tibial fractures remains debated, it may improve restoration of length and alignment in selected fracture patterns, particularly when the associated fibular fracture contributes to instability [11-13]. In our series, supplementary fibular plating may have helped improve overall construct balance and reduction control, especially in the setting of distal metaphyseal fracture geometry [11-13].

Radiographic progression in this series was also consistent with the biological principles of MIPO. Early follow-up images showed maintained reduction and callus formation, while later radiographs confirmed union and remodeling. Functional recovery was favorable as well, with good to excellent AOFAS ankle-hindfoot scores in the cases where formal scoring was available, along with satisfactory walking ability and ankle function across the series. These findings are in keeping with previous reports describing reliable healing and useful functional recovery after minimally invasive plating of selected distal tibial fractures [5-10].

From a technical perspective, several factors likely contributed to the observed outcomes: careful case selection, preservation of fracture biology, indirect reduction, stable fixation with a distal tibial locking construct, and protection of soft tissues through limited exposure [4-6]. These points deserve emphasis because successful MIPO depends not only on implant choice but also on adherence to biological fixation principles and meticulous attention to alignment [4-6,11].

This study has limitations. It is a small retrospective case series without a control group, so direct comparison with intramedullary nailing or open reduction and internal fixation cannot be made. In addition, follow-up intervals were not fully uniform, and in Case 3, the precise timing of radiological union could not be determined because serial radiographs were not obtained at standardized time points. Nevertheless, later imaging clearly demonstrated complete union with remodeling.

Despite these limitations, the consistent fracture healing, maintenance of alignment, absence of major complications, and satisfactory functional outcomes across all three cases support the role of MIPO as an effective treatment option for selected extra-articular distal tibial fractures. This series suggests that early minimally invasive plating, combined with respect for fracture biology and preservation of the soft-tissue envelope, can produce reliable clinical and radiographic outcomes in AO/OTA 43-A1.2 distal tibial fractures.

## Conclusions

MIPO is an effective and reliable treatment option for selected extra-articular distal tibial fractures. In this case series, early biological fixation with preservation of soft tissues resulted in consistent fracture union, maintenance of alignment, and satisfactory functional outcomes without major complications. Careful patient selection, adherence to biological fixation principles, and meticulous surgical technique are essential to achieve optimal clinical and radiographic results.
